# Scenario-Led Habitat Modelling of Land Use Change Impacts on Key Species

**DOI:** 10.1371/journal.pone.0142477

**Published:** 2015-11-16

**Authors:** Matthew Geary, Alan H. Fielding, Philip J. K. McGowan, Stuart J. Marsden

**Affiliations:** 1 Division of Biology & Conservation Ecology, School of Science & the Environment, Manchester Metropolitan University, Manchester, United Kingdom; 2 World Pheasant Association, Newcastle University Biology Field Station, Close House Estate, Heddon-on-the-Wall, United Kingdom; University of Maryland at College Park, UNITED STATES

## Abstract

Accurate predictions of the impacts of future land use change on species of conservation concern can help to inform policy-makers and improve conservation measures. If predictions are spatially explicit, predicted consequences of likely land use changes could be accessible to land managers at a scale relevant to their working landscape. We introduce a method, based on open source software, which integrates habitat suitability modelling with scenario-building, and illustrate its use by investigating the effects of alternative land use change scenarios on landscape suitability for black grouse *Tetrao tetrix*. Expert opinion was used to construct five near-future (twenty years) scenarios for the 800 km^2^ study site in upland Scotland. For each scenario, the cover of different land use types was altered by 5–30% from 20 random starting locations and changes in habitat suitability assessed by projecting a MaxEnt suitability model onto each simulated landscape. A scenario converting grazed land to moorland and open forestry was the most beneficial for black grouse, and ‘increased grazing’ (the opposite conversion) the most detrimental. Positioning of new landscape blocks was shown to be important in some situations. Increasing the area of open-canopy forestry caused a proportional decrease in suitability, but suitability gains for the ‘reduced grazing’ scenario were nonlinear. ‘Scenario-led’ landscape simulation models can be applied in assessments of the impacts of land use change both on individual species and also on diversity and community measures, or ecosystem services. A next step would be to include landscape configuration more explicitly in the simulation models, both to make them more realistic, and to examine the effects of habitat placement more thoroughly. In this example, the recommended policy would be incentives on grazing reduction to benefit black grouse.

## Introduction

Land use change has been shown to be an important driver of population change in a wide variety of taxa [1], with changes such as conversion of forest to agricultural land reducing habitat availability for species throughout the world [2]. Even subtle changes to the way the landscape is managed, such as the timing of ploughing cereal fields [3], can have a significant impact on the ecology of wildlife within an area [4]. Working landscapes, such as farmland or commercial forests, are often compromises between the interests of biodiversity preservation and economic benefit [5]. How much biodiversity persists within working landscapes, of course, varies widely, but, with careful planning, wildlife can thrive alongside human land use. For example, an area in Oregon, USA, retained around 97% of the biological value while still maintaining 92% of its economic value [6].

The Scottish uplands are a working landscape containing a mosaic of grouse moor, deciduous and coniferous woodland, and pasture [7]. The composition of the landscape is, of course, dynamic, with grant schemes such as the Scottish Rural Development Programme [8] influencing the amount and placement of various land use types. With these changes come changes in wildlife populations, some species being seriously affected [9,10]. One such species is the black grouse *Tetrao tetrix*, which experienced serious declines throughout the United Kingdom during the twentieth century [11] but which is expected to benefit from land use change brought about through both payments to land-owners and wind farm habitat management plans [8]. Black grouse is a bird of woodland edges and requires a habitat mosaic which can consist of mixtures of moorland, forest and agricultural land [12,13], features which have been altered by land use changes in the UK uplands over the past decades[14].

Modelling species distributions in terms of their associations with habitats, land use or environmental factors is a rapidly advancing area of ecological research [15]. Many applications of these models have been static, assessing species distributions at a 'snapshot' in time [16]. Static species distribution modelling can inform conservationists about current [17] and potential distributions [18] or population connectivity [19]. A logical extension is to project organism-environment relationships into the future for example, to predict species distributions under climate change scenarios [20]. Informed environmental policy can be of great benefit to conservation [21,22] but the challenge for ecologists is to maximise the relevance and accessibility of conservation research to policy-makers. To this end, it is important that the projection of species distributions onto future landscape scenarios is ecologically realistic, and therefore, restricts the projection to the shorter term and expand species distribution models to include potential habitat-management or land use changes. Scenario-led models allow the potential impacts of policy or conservation action to be quantified and compared [6,23].

Here we demonstrate the use of open source software to simulate land use change in the Scottish uplands and its effect on black grouse. We developed five land use change scenarios: reduced grazing, increased grazing, increased grouse moor (heather moorland actively managed to provide high red grouse *Lagopus lagopus* densities for driven shooting), increased closed-canopy forestry and increased open-canopy forestry to reflect potential land use changes in the study area which may have a positive or negative impact on the species [14,24]. We ‘grew’ patches of the new habitat, using cellular automata according to the land use change scenarios, in randomly chosen positions to produce a range of estimates for the impact of the land use change on black grouse over multiple runs. We then examined the effects of the landscape changes in more detail by looking for nonlinearities in response as the land use change became more extensive. Finally, we discuss the potential that scenario-led landscape simulation modelling has as a practical tool for policy-makers interested in integrating conservation objectives and land use policy.

## Materials and Methods

### Study area and lek location data

The study area is 800 km^2^ around Loch Tummel in Perthshire, Scotland (56°42′40″N 3°55′40″W). Altitude ranges from 46 to 1078 m above sea level. Forestry, agriculture and moorland managed for shooting game (generally hunting of red deer *Cervus elaphus* and red grouse) are the main land uses in the area which is representative of black grouse habitat within working landscapes in much of Scotland. In order to demonstrate our modelling method, we required good quality data for both the location of black grouse display sites (*i*.*e*. leks; areas at which male black grouse display in order to obtain access to females) along with a clear satellite image on which to base a habitat map. With this in mind, rather than demonstrating the modelling approach with a lower quality satellite image and the most recently available black grouse data we chose instead to base these simulations on 1994, a year in which the black grouse population was relatively large (663 displaying males compared with a mean number of 353 displaying males over the next 15 years), was thought to have been counted fully and for which a clear Landsat image was available.

Lek locations used in this study were provided by Perthshire Black Grouse Study Group (PBGSG). The PBGSG is a group of volunteers, including core members from professional bodies including the Game and Wildlife Conservation Trust (GWCT), Royal Society for the Protection of Birds (RSPB) and Forestry Commission. A core of a few volunteers surveyed large areas for several years (e.g. Forestry Commission land) while a number of volunteers surveyed smaller areas in just a few years. These data represent one of many years of a long-term study undertaken by PBGSG (1990–2008) and which used methods similar to those used in the two national black grouse surveys [11,25] covering as much of the landscape as possible rather than using transect methods. Volunteers walked within 0.5 km of all suitable habitat [26] while listening for male calls to locate lek sites. These sites were subsequently visited twice more, each up to two hours after dawn, to verify the presence of a lek and to count the number of displaying males [25]. As the purpose of the survey was to count the number of displaying males at existing leks, absences were not recorded systematically. Areas above 550 m (18% of the study area) were considered unsuitable for black grouse leks by Hancock et al. [25] and so were not searched by PBGSG, and nor were dense forestry, built up areas and arable farmland. Surveys usually focus attention on likely areas to search so as not to spend effort in areas known to be outside the range for the target species. This was the case for the black grouse surveys which excluded areas above 550 m. However, below that altitude, there is still much local heterogeneity in habitat suitability for black grouse [24] which is what the survey method attempted to capture [26].

### Environmental data and habitat suitability modelling

National land cover maps are available for the UK but these have received criticism, particularly with respect to their inability to accurately identify upland habitat types [27,28]. It therefore made sense to develop our own habitat groupings based on unsupervised classification coupled with a good working knowledge of the study area. Habitat data were taken from the USGS LANDSAT image at 30 m resolution (path 206, row 20) from 1994; as the image for this region was clear, atmospheric correction was not applied. The original image consisted of eight spectral bands of which six were combined using principal components analysis (PCA; in ArcMap 9.2), chosen as the most straightforward method in the available software, to remove redundant information. The thermal infra-red band and the panchromatic band were omitted from the classification. The processed image, consisting of three principal components, was classified into habitat types using an unsupervised classification in Multispec [29]. As the satellite image was from 1994, contemporaneous land cover information was unavailable; therefore unsupervised classification was preferred [30]. The k-means algorithm is a simple and efficient algorithm which has two steps that are repeated (iterated) until an optimisation is reached [31]. Initially the data (pixels) are partitioned into a defined number of clusters, usually at random. Cluster centroids ('averages') are calculated and each case is moved to its most similar cluster centre. The cluster centres are recalculated and cases are again moved. These steps are repeated, leading to increasingly homogeneous clusters, until an endpoint is reached. Many habitats, particularly woodland, agricultural grasslands and large anthropogenic features are clearly identifiable on a false composite colour image of the satellite scene, and these are correctly represented in the resulting automated classification. This classification resulted in 18 habitat types which were subsequently pooled into six broad categories using detailed knowledge of the study area, stakeholder interviews, field visits, and more recent (2005 & 2012) aerial photography as a reference. This photography was used to confirm features which were likely to have remained fixed during this period (e.g. plantation forest edges, water bodies and field boundaries) and was used in conjunction with the other methods of verifying the landscape classification and tested with comparison to an existing land cover map from 1990 (S1 File). These broad categories were: human-dominated landscapes (buildings, urban areas, roads and roadside verges); grazed land (managed enclosed grasslands with relatively productive grasses, as well as open grazed land dominated by rough, poor quality grassland such as *Nardus* and *Molinia*); moorland (open land usually dominated by heather *Calluna vulgaris*); open-canopy forestry; closed-canopy forestry; and water bodies. Proportions of each habitat type within a 2 km radius of each grid cell were calculated using the focal command in the 'raster' package [32] in R [33]. A radius of 2 km was considered to give a conservative estimate of the territory used by individuals throughout the year [34,35]. This proof of concept study, as with many studies on black grouse, was based on lek location positions although it could be repeated or extended by using feeding locations, or locations of birds at particular life stages or in different seasons. A raster giving the altitude of each grid cell was obtained from Ordnance Survey (Ordnance Survey data: OS Landform PROFILE; 50 m resolution), then cropped and converted to the same resolution (28.5 m) and extent as the habitat maps.

Habitat suitability modelling was performed using MaxEnt [36] within the 'dismo' package [37] in R using seven predictors (the proportion of six habitat types plus altitude). MaxEnt is a presence-only, machine learning process and has been shown to outperform other presence-only habitat suitability modelling methods [36,38]. MaxEnt produces values for the relative suitability of each pixel of a map (i.e. relative to the rest of the landscape used in the model rather than probability of presence [39]). We tested a range of values for the regularization parameter (β) within our models (values of 1, 2, 3, 5, 7, 9, 10, 11, 13, 15, 17, and 19) and selected the ‘best’ value for our model using the value with the lowest AIC score [40,41]. Default settings were used for all other variables within our model fitting 10 crossvalidated replicates [24]. Full details of the MaxEnt model used to assess the simulated landscapes in terms of habitat suitability for black grouse can be found in the supplementary information (S2 File).

To compare changes in landscape suitability after modelling with this base map, we converted the relative suitability predictions into a binary, presence/absence prediction for black grouse across the landscape using a habitat suitability threshold. The choice of threshold is extremely important [42] and in some cases can be a complex and case-specific choice. To keep these choices relatively simple for demonstration purposes, in this case, we tested three thresholds. These were low, medium and high suitability, corresponding to the first quartile, median and third quartile relative suitability scores, based on a habitat suitability model for the original habitat in 1994.

The most common metrics used to assess the predictive power of species distribution models require both presence and absence values for testing. Tests of predictive power were calculated from our presence-only dataset by producing a composite dataset consisting of our original presence points along with 1000 background predictions from the binary presence/absence map similar to the map described above. This was created using a fourth threshold, the maximum sum of sensitivity and specificity. The MaxEnt model based on the original habitat was then tested using the area under the curve (AUC, ranges from 0 to 1) of the receiver operating characteristic plot (ROC [43]) as well as the true skill statistic (TSS, ranges from -1 to +1 [44]). In both cases, the closer the value is to 1, the more accurate the model.

### Scenario building and landscape change simulation

The study area represents a highly dynamic working landscape. Analysis of satellite images from 1994 and 2008 show that while net gains or losses in each habitat type between 1994 and 2008 didn’t exceed 3% for any major habitat type, the amount of land actually switching between habitat types was considerably larger (up to 17%; indicating both gains and losses in different parts of the landscape). During this period the black grouse population experienced a significant decline, followed by a recovery. Changes in habitat over this time and the impact on black grouse populations are explored in Geary *et al*. [45]. In order to choose appropriate future land use change scenarios for this proof of concept study, expert advice was sought. A questionnaire prompted respondents to choose their top five from ten potential scenarios, these coming from the authors' experience in upland research and the likelihood that they would take place during the next twenty years in Scotland. Surveys were received from ten professionals representing academic (30%), consultant (10%), conservation (30%), governmental (20%) and sporting interests (10%). The most likely future scenario was considered to be additional native forestry schemes resulting in more open-canopy forestry (Table 1). The second to fifth most likely scenarios were an increase in grouse moor, a decrease in grazing, an increase in grazing and an increase in closed-canopy forestry. Agreement among scenarios was generally good with each of the scenarios used in modelling chosen by over 50% of the experts (S3 File).

**Table 1:** Scenarios used in landscape simulation modelling. Scenarios used in landscape simulation modelling, along with how the landscape is changed under each. Scenarios were chosen by upland experts as most likely to occur in the Scottish uplands from ten candidate scenarios. The ranked likelihood of each scenario, as decided by respondents to the questionnaire is also presented.

SCENARIO	RANK	CHANGES TO THE LANDSCAPE
Increased open-canopy forestry	1	Woodland creation grants larger than those for plantation forestry are available for the planting of native forestry under the (SRDP; http://www.scotland.gov.uk/Topics/farmingrural/SRDP/RuralPriorities/Options/). This will be reflected by a conversion of 7% of grazed land and 3% of grouse moor to open-canopy/mixed woodland. Grazed land was considered more likely to be converted to woodland than grouse moor.
Reduced grazing	2	Since 1982, sheep numbers in Scotland have decreased by 34% [70]. Grants encouraging a reduction in grazing are currently available through the Scottish Rural Development Programme (SRDP; http://www.scotland.gov.uk/Topics/farmingrural/SRDP/RuralPriorities/Options/). Under this scenario, a continued reduction in upland grazing will be reflected by a 10% reduction in grazed land 7% of which will be converted into moorland and 3% into open-canopy/mixed forestry.
Increased grouse moor	3	Economic analysis of the grouse shooting industry by the Fraser of Allander Institute [71] showed increased profitability in managed grouse shoots in 2010 and suggested this may lead to an increase in the area of moorland used for shooting. This will be reflected by a conversion of 5% of grazed land and 5% of open-canopy woodland to grouse moor.
Increased grazing	4	Both the Tenant farmers association and the Pack enquiry [72] have suggested a return to headage payments for upland farmers. In the past, this has resulted in an increase in the number of sheep. This change will be reflected by converting 10% of moorland to grazed land.
Increased closed-canopy forestry	5	Woodland creation grants available under the (SRDP; http://www.scotland.gov.uk/Topics/farmingrural/SRDP/RuralPriorities/Options/) offer a financial incentive for the creation of plantation forestry. This will be reflected in the conversion of 5% of grouse moor and 5% of grazed land to plantation forestry.

For each scenario, land use change was simulated from 20 random starting locations (i.e. 20 random pixels from the 1994 classified image) using an iterative process which grows new pixels of habitat close to starting locations according to a probability of change (0.25; simply to determine the direction in which the patch grows) until they reached the new proportion of the habitat prescribed under the scenario. Starting locations refer to individual pixels around which simulated land use change was centred and do not imply likely land use change at that point to be greater than at any other. The resulting habitat patches were non-uniform in shape and, due to the stochastic process, varied in size, but their combined area summed to the total area of new habitat. With the new habitat patches created, the proportion of each habitat type within 2 km of each grid cell in the study area was re-calculated. The 1994 MaxEnt model was then projected onto these novel landscapes and the percentage of the study area predicted as suitable habitat for black grouse was calculated for each threshold suitability value. R scripts for the modelling functions as well as the scenarios are included in the supplementary information (S4 File). Thirty new landscapes (i.e. 30 model runs) were generated for each scenario to allow average habitat suitability to be calculated. Results from these new scenarios could potentially have been influenced by changes in the spatial arrangement of patches rather than those produced by the actual increase or decrease in the different habitat types. To explore this further we created a series of ‘null’ models for each scenario to compare situations with land use change against scenarios where the habitat is changed in the same way but with no net increase or decrease in the amount of each habitat type. Again, thirty new landscapes were generated for each of four null models (both increase in grazing and decrease in grazing have the same null model–no net change in grazing) and the 1994 MaxEnt model was then projected onto them, and the percentage of the study area predicted suitable for black grouse calculated.

The proportions of area predicted suitable under each of the five scenarios were compared using Kruskal-Wallis tests followed by pairwise Mann-Whitney U tests. In addition, each scenario was compared to its equivalent ‘null’ scenario using Mann-Whitney U tests. We examined in greater detail the impact of some closed-canopy forestry patches, a common land use change in the Scottish uplands, as they are thought to be detrimental for black grouse at a landscape scale [14] but changed land management around them could produce complex effects on habitat suitability. To do this, we present some examples of the effects of different placements of closed-canopy forest patches within the landscape. Supporting these examples, we present pre- and post-scenario landscape metrics such as number of closed-canopy forestry patches, mean patch sizes and total woodland edge computed using the SDMTools package [46]. Further to this, we investigated whether there was a linear (proportional) effect of adding increasing amounts of the given land use on habitat suitability. We varied the area affected by increased open-canopy forestry and reduced grazing, two land use changes which are thought to benefit black grouse [47], between 5% and 30% to identify any nonlinearities in benefit for black grouse.

While we use real data on lek presence for black grouse to illustrate our method, we do not include any demographic data in the study. As well as the suitability of habitat surrounding the lek we acknowledge that other considerations such as lek connectivity [48], the quality of habitat around surrounding leks [24], edge effects and source-sink dynamics are likely to also play a role in determining the overall quality of the landscape for this species. Although the model presented retains enough flexibility to incorporate these considerations, we have chosen to focus solely on habitat quality for the sake of simplicity while demonstrating the method.

## Results

### Comparisons across scenarios

**Fig 1:**
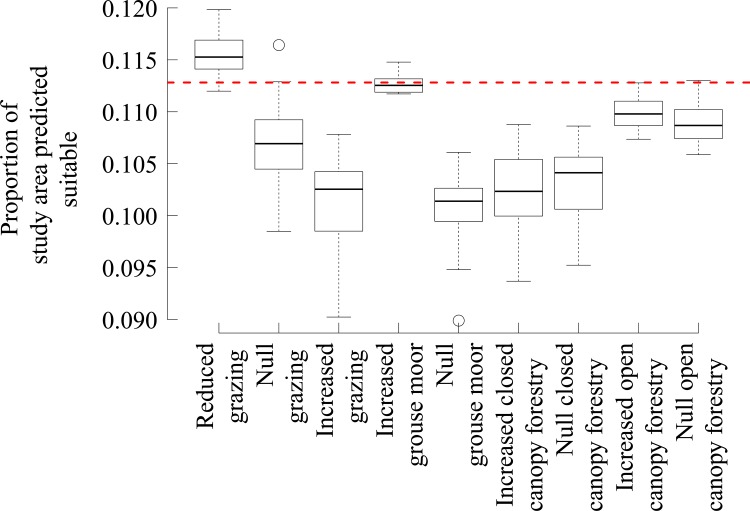
The proportion of the study area predicted suitable for black grouse under each of the land use change scenarios and their ‘null’ equivalent. Boxplots of the proportion of the study area predicted as suitable for black grouse using the median threshold (0.58) under each land use change scenario along with its ‘null’ equivalent where the configuration of land uses was changed but not the proportion of each land use type. The dotted line indicates the proportion of the study area predicted suitable using the original habitat.

**Fig 2:**
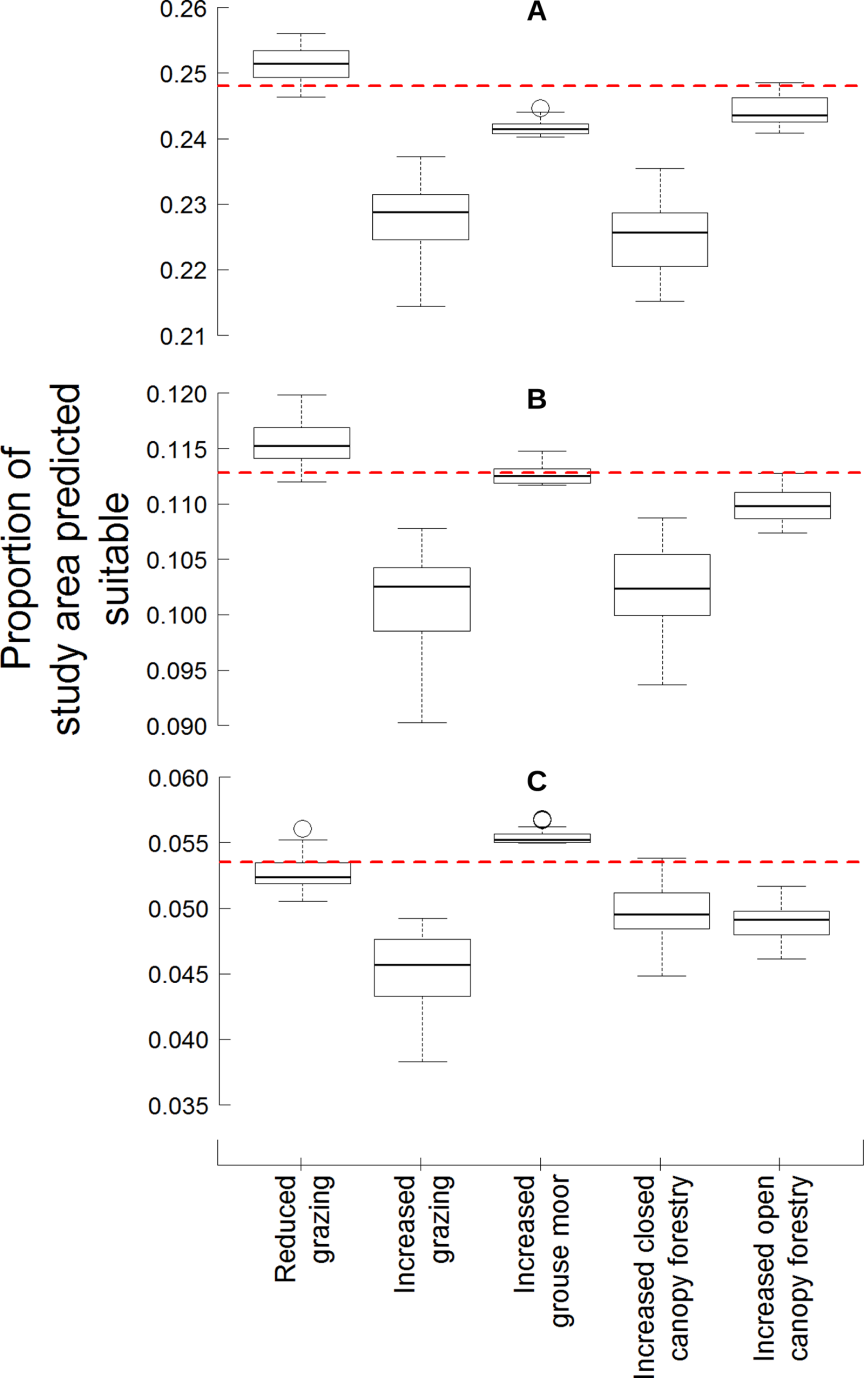
The proportion of the study area predicted suitable for black grouse under each of the land use change scenarios. Boxplots of the proportion of the study area predicted as suitable for black grouse using the A) lower quartile (0.48), B) median (0.58); and, C) upper quartile (0.65) thresholds using 30 m pixels. The dotted line indicates the proportion of the study area predicted suitable using the original habitat at each threshold.

The MaxEnt model, using a regularisation multiplier of 7 (Table 2), performed well in predicting black grouse presence using the original environmental data (30 m pixels, AUC = 0.83, TSS = 0.64). All of the scenarios produced outcomes that were significantly different from those of the ‘null’ scenarios (Fig 1) at each of the three thresholds (min U = 0, n = 30, max *P* = 0.04) except for the open canopy forestry scenario which was not significantly different at the first and third quartile thresholds (min U = 481, n = 30, min *P* = 0.06) and the closed canopy forestry scenario which was not significantly different from the null scenario at using the median threshold (W = 390, n = 30, *P =* 0.38). There were significant differences between the amount of suitable habitat produced under the five scenarios at the first quartile (χ^2^ = 132.6, df = 4, *P* < 0.001; Fig 2A), median (χ^2^ = 129.6, df = 4, *P* < 0.001; Fig 2B) and third quartile (χ^2^ = 120, df = 4, *P* < 0.001; Fig 2C) thresholds. Across the three thresholds the most beneficial scenario for black grouse in terms of increased habitat suitability across the landscape was the reduced grazing scenario. It resulted in a significantly larger proportion of the landscape suitable for black grouse than the next best scenario at the first quartile (increased open canopy forestry; U = 900, n = 60, *P* < 0.001) and median (increased grouse moor; U = 817, n = 60, *P* < 0.001) thresholds. At the third quartile threshold increasing grouse moor resulted in a significantly larger proportion of the landscape predicted suitable for black grouse than the next best scenarios (reduced grazing; U = 51, n = 60, p < 0.001). The lowest suitability at the first and third quartile thresholds was produced by the increased grazing scenario. This predicted significantly smaller proportions of the landscape were suitable for black grouse than the next lowest scenario at both thresholds (Q1 closed canopy forestry; U = 607, n = 60, *P* = 0.02, Q3 open canopy forestry; U = 107, n = 60, *P* < 0.001). Using the median threshold, the scenario predicting the lowest suitability was increased closed canopy forestry. However, this did not predict significantly lower suitability than the next lowest scenario (increased grazing; U = 406, n = 60, *P* = 0.52). Both the increased closed canopy forestry (U = 2, n = 60, *P* < 0.001) and increased grazing (U = 18, n = 60, *P* < 0.001) scenarios resulted in significantly lower suitability for black grouse than the next worst scenario, increased open canopy forestry.

**Table 2:** Model selection comparing regularisation parameters (β) in candidate MaxEnt models.

Regularisation parameter (β)	Log Likelihood	Parameters	AICc score	ΔAICc
1	-946.569	37	2052.35	111.38
2	-953.742	19	1960.39	19.41
3	-958.848	16	1959.77	18.80
5	-966.734	13	1965.85	24.88
7	-958.653	10	1940.97	0
9	-978.316	8	1974.95	33.98
10	-980.113	9	1981.18	40.20
11	-983.429	9	1987.81	46.84
13	-985.944	8	1990.21	49.24
15	-987.984	6	1989.28	48.31
17	-988.778	6	1990.87	49.90
19	-990.25	6	1993.81	52.84

### Effect of habitat placement

**Fig 3:**
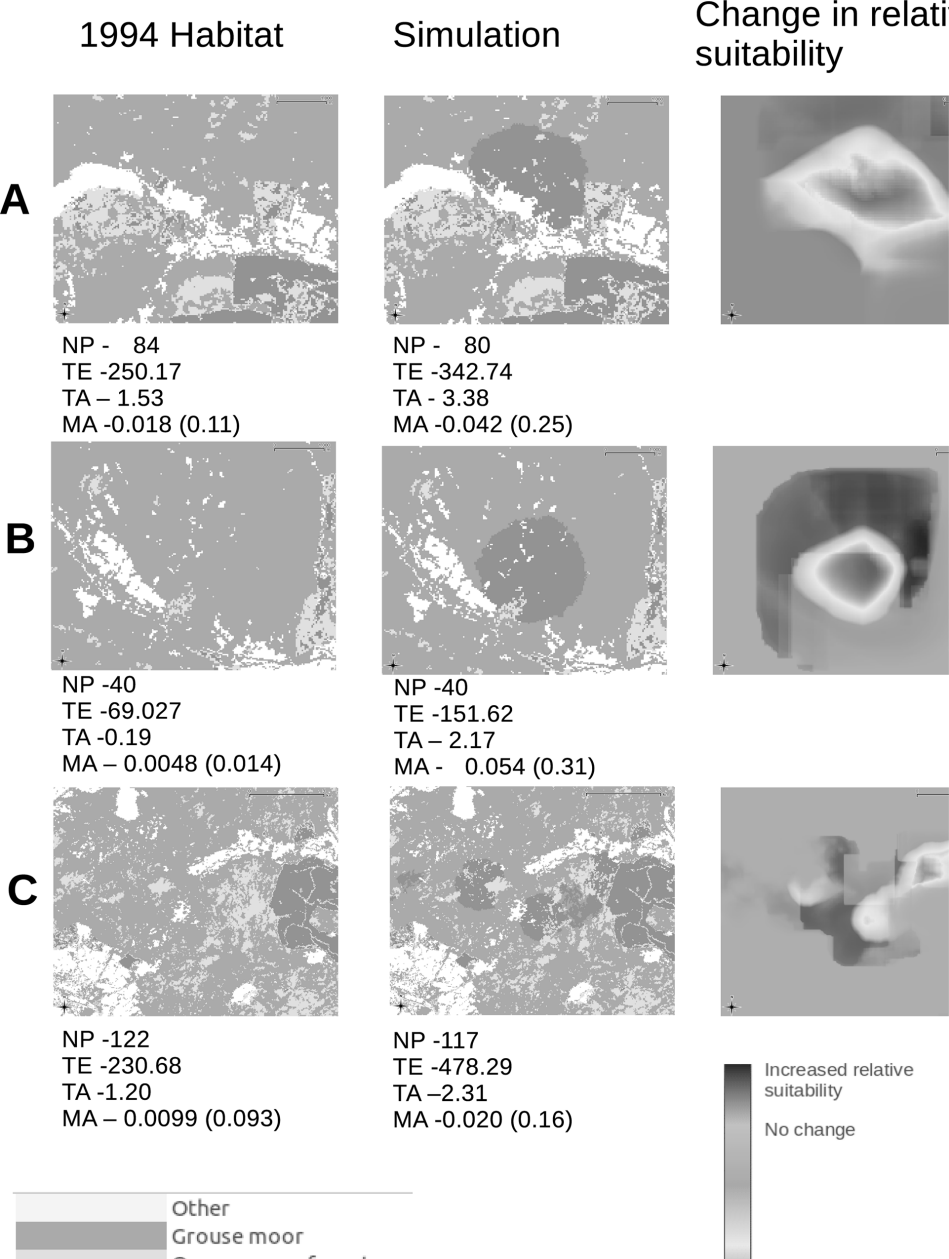
Examples of different outcomes resulting from closed canopy forestry placement within the landscape. Examples of new closed-canopy forestry placement resulting in A) a reduction in habitat suitability within the new forest, B) a decrease in habitat suitability within the new forest coupled with an increase in habitat suitability around the new forest edge, and, C) a reduction of habitat suitability within the new forest with an increase in habitat suitability around some of the forest edge. Text under individual habitat examples shows NP–Number of forestry patches, TE–Total forest edge (km), TA–total forest area (km^2^) and MA–mean forest area (km^2^).

Changes to the area and position of closed-canopy forestry resulted in projections which could both increase and decrease suitability for black grouse. Fig 3A–3C represent simplified small sections of the landscape showing 1994 habitat and altered habitat under an illustrative ‘one-off’ simulated land use change, along with resultant differences in suitability. These figures are presented for reference only as further analysis of this effect is beyond the scope of this paper. Below each map are selected landscape metrics such as number of closed-canopy forestry patches, mean patch sizes and total woodland edge. In most cases, the area covered by new forestry was much less suitable (Fig 3A), but in some situations, the area immediately surrounding the new forest had improved suitability (Fig 3B). In other areas, a more complex arrangement arose where habitat suitability had increased along some edges but not others (Fig 3C).

### Effects of extent of land use change

**Fig 4:**
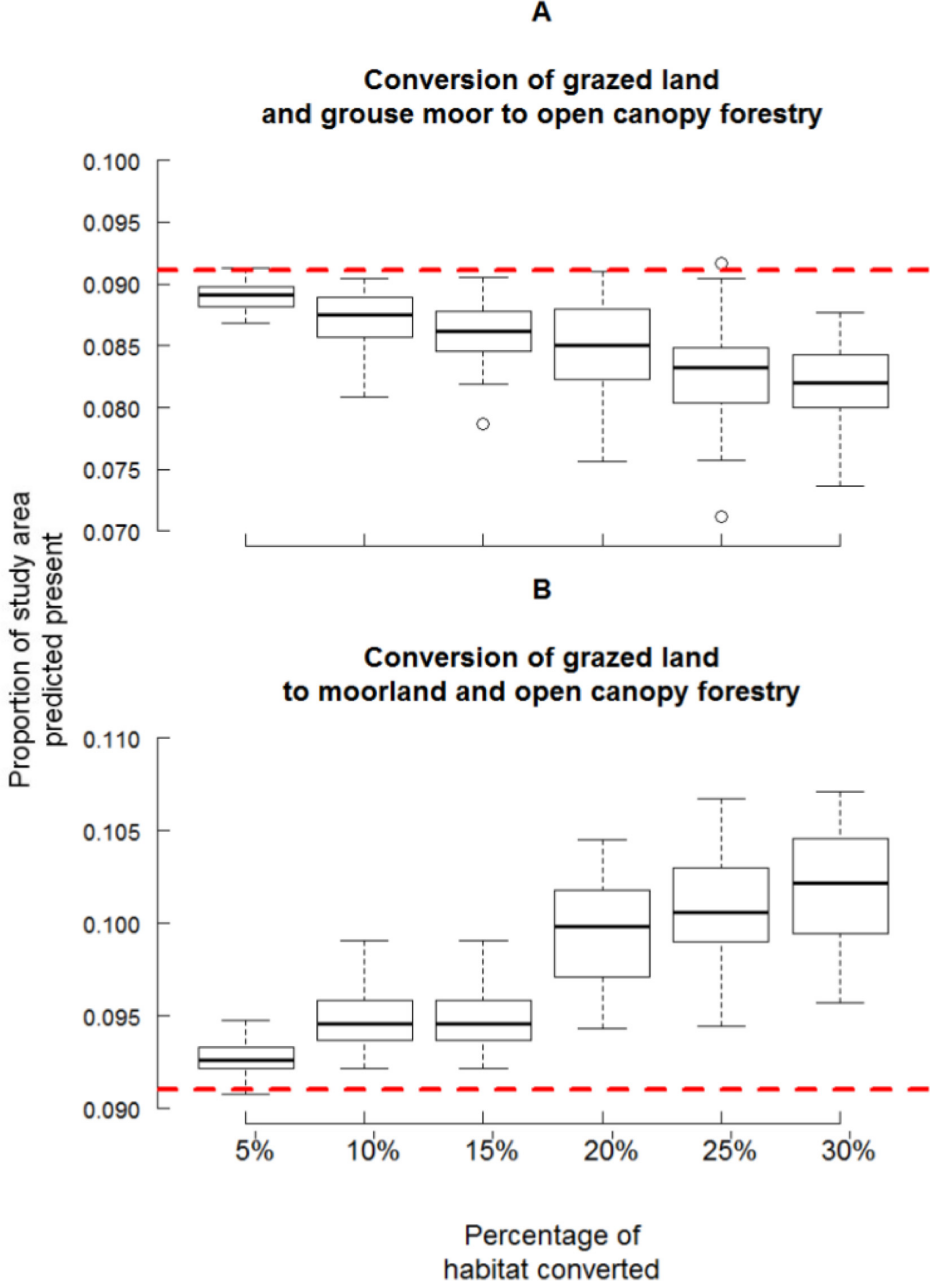
The proportion of the study area predicted suitable for black grouse under different levels of habitat change. Boxplots representing the proportion of the study area predicted present under scenarios different levels of habitat change (5–30%) using the median threshold (0.58) for A) the increased open-canopy forestry scenario, and, B) the reduced grazing scenario. The dotted line represents the proportion of the study area predicted suitable using the original habitat.

Increases in the extent of open-canopy forestry resulted in a linear decrease in suitability (Fig 4A). For the reduced grazing scenario, increasing the extent of land use change resulted in a nonlinear increase in suitability (Fig 4B) which was disproportionately beneficial when 20% or more of grazed land was converted (median 15% = 0.095 median 20% = 0.1; U = 124, n = 100, *P* < 0.001).

## Discussion

Fitting with our expectation, the most beneficial potential future scenario for the black grouse involved the conversion of grazed land to both grouse moor and open-canopy forestry and the most detrimental was an increase in grazing. This result reflected the detrimental impact of overgrazing on black grouse as well as their habitat requirements as woodland-edge species [49,50] although this demonstration of our method does not yet take into account fragmentation or the influence of habitat edge independent of habitat area. Consideration of changes to the amount of edge would be useful to include in future models, especially for species such as black grouse, as it would help to properly reflect patch shapes as well as sizes, especially in heavily-fragmented landscapes. Sheep grazing is a widespread feature of landscapes in the Scottish uplands and with added pressure from large red deer populations [51], overgrazing is a threat to many upland bird species [9]. Reduced grazing regimes, both in terms of fewer sheep and increased deer control, are likely to have widespread benefits for a number of species of conservation concern [52], including black grouse [50,53]. The numerical predictions of changes to the area suitable for black grouse should be regarded relative to the other scenarios rather than suggestions of the actual changes ‘on the ground’.

Under the increased closed-canopy forestry scenario, different model runs produced both increases and reductions in habitat suitability. Research has shown that canopy closure in areas of new plantation forestry has led to declines in black grouse populations [14]. Our example simulations using increased closed-canopy forestry (Fig 3) showed that the location of new patches of closed-canopy forestry was important in determining the resulting suitability for black grouse. That there is a landscape context to the effects of land use change on species is not surprising [54], especially in a species such as black grouse, which is associated with habitat mosaics [12,13], and which can thrive in several rather different combinations of land uses [13,24,55]. Indeed in our null models, we quantified the variability in habitat suitability associated with moving habitat patches around rather than actually changing than proportions of habitats. We then briefly explored some of the possible metrics, such as patch size and edge effects, which may affect the impact of different patch placements on overall landscape suitability, but it will take a more extensive modelling exercise to elucidate the multiple features that contribute to landscape suitability. Our proposed method does, however, allow quantification of suitability changes related to individual landscape changes, even if it does not identify the underlying causes of those changes. At present, our scenarios are restricted to single land use changes (although an increase in one land use results in loss of one or more other land uses) for ease of model demonstration. In reality, changes may involve several land use types, as well as gradual changes in habitat characteristics brought about natural habitat succession (e.g. canopy closure) and management interventions (e.g. heather burning). Taken together with landscape context, this complexity has the potential to become prohibitively computing-intensive to model accurately [56]. Again, such models make for powerful ecological tools, and our illustration is a first practical step towards their realisation. As expected, the interiors of closed-canopy plantations were consistently unsuitable [14,47], although the same was not true for the areas surrounding new forest patches. Forest edges are a habitat feature preferred by black grouse [49] and investigating the effect of patch shape on habitat suitability may be productive for land managers.

At their most basic, increases or decreases in suitability would be proportionate to the amount of habitat change (e.g. stone marten *Martes foina* and strawberry tree *Arbutus undo* [57]) but more complex, nonlinear relationships, perhaps depending on landscape structure or interacting effects (e.g. models of invasive species [58]) or ecological thresholds (e.g. pine marten *Martes martes* in fragmented forests [59]) are important in wildlife management. Increases in the area of grazed land converted to grouse moor and open-canopy forestry was beneficial to black grouse, with a step change at proportions above 15%. The spatial structure of the resulting landscapes is a likely cause for this difference in response [58]. In one case, both moorland and grazed land were converted into open-canopy forestry causing a homogenisation of the landscape which larger magnitudes of change exacerbated. In contrast, reduced grazing resulted in a more heterogeneous landscape which contained the mosaics attractive to black grouse [48,51]. Nonlinear responses to habitat management by species can be related to edge effects [60]; as a forest edge species [49], black grouse might benefit from these changes. Identifying these thresholds is important for species conservation as it highlights the potential for rapid changes in abundance or distribution to occur [61,62].

Combined with knowledge of habitat management, scenario-led habitat suitability modelling could be extremely useful both for agencies or consultants advising individual landowners on local costs/benefits of land management changes [63], and as a basis for encouraging wider-scale changes through appropriate policy or planning regulations [64]. Changes incorporated into the models could be the results of changes in policy across landscapes, as we have demonstrated here, or specific spatial changes of interest to land managers. Species of conservation concern which inhabit agricultural landscapes could benefit greatly from changes to management practices (e.g. corn bunting *Miliaria calandra* [65]) or from subsidies targeted to improving habitats (e.g. little bustard *Tetrax tetrax* [66] & greater sage-grouse *Centrocercus urophasianus* [67]) both of which could be explored using landscape simulation models. A natural progression would be to consider the position and shape of new land use features as well as their location within the landscape. This would require an extension of the current modelling framework and consideration of the computational requirements of creating realistic representations of these complex situations. Landscape simulation models could be extended to work on metrics such as species richness/diversity, or ecosystem function or services (e.g. abundance of pollinators [68]). If the provision of ecosystem services or economic benefits is related to habitats or landscape structure then, by examining the effects of different land use change scenarios, a compromise between economic activity and conservation can be achieved [6]. The inclusion of economic or sociological factors into landscape simulation models is another area of potential, with, for example, likelihood of land use change related to an ‘index of willingness’ which might be affected by differing financial rewards or levels of knowledge [69].

Black grouse in the Scottish uplands, like many species across the world, now exist largely within a working landscape [47]. Our models pointed to specific land use changes which are predicted to improve habitat for black grouse, finding a reduction in grazing, as field-based studies have [53], to be the most beneficial. Perhaps most importantly, our work indicates that it is not just the land use change itself that will determine whether species thrive or decline, but the extent of these changes and their position in relation to other features in the landscape.

## Supporting Information

S1 File Validation of the habitat classification. Validation of our own habitat classification with reference to the UK national land use classification.
(DOC)

S2 File Modelling black grouse habitat suitability using MaxEnt. A description on the MaxEnt model used to assess habitat suitability under each scenario.
(DOC)

S3 File Scenarios used in landscape simulation modelling. A description of the potential scenarios indicating the scenarios chosen by experts as the most likely and the agreement between choices.
(DOCX)

S4 File R code for landscape simulation modelling. R code to run the models under the stated scenarios.
(R) Click here for additional data file.
