# Supplementary material for: Scenario-Led Habitat Modelling of Land Use Change Impacts on Key Species
Source: PLoS One. 2015 Nov 16;10(11):e0142477. doi: 10.1371/journal.pone.0142477 (PMC4646449; doi:10.1371/journal.pone.0142477)
Supplement: S1 File — Validation of our own habitat classification with reference to the UK national land use classification. (DOC) [file pone.0142477.s001.doc]

**Appendix I – Validation of habitat classification**

In order for the lek locations and habitat variables used to predict habitat suitability for black grouse to be from the same year, we chose to use lek records from 1994 when there was a clear Landsat image of the study area available. Land cover maps of the British Isles are available for 1990 and 2000, however, the lek surveys in our study area began in 1990 and there is evidence that during this year not all leks were found and the population in this region was at its lowest in 2000 giving fewer presence locations than would be optimum for modelling. In using our own habitat classifications, and especially using an unsupervised classification procedure, it is important to test the accuracy of our land cover maps. To this end we compared our habitat locations to LCM 1990 (http://www.ceh.ac.uk/landcovermap1990.html), a widely used map of UK land cover. All analyses were performed in R (R Development Core Team 2012) .

*Resolution*

We used the LCM 1990 dominant land cover map at a 1 km resolution. Our habitat data were at a resolution of 28.5 m so our maps were scaled up by the modal habitat value in each 1 km grid cell. As this re-projection resulted in slight differences in the number of rows and columns in the raster maps the LCM 1990 map was re-sampled using a nearest neighbour method to match the extent and size of the Landsat derived map.

*Conversion of LCM1990 land cover categories*

LCM 1990 has 18 land cover categories whereas our simplified classified maps for modelling have only six. To compare between the two it was necessary to reclassify LCM 1990 into our own categories. This was done by placing each LCM 1990 category into one of our own based on the description of the land cover in question (Table 1.).

**Table 1.** Re-classification of LCM 1990 into our six land cover categories

| **This study** | **LCM 1990** |
| --- | --- |
| *Human-dominated areas* | 18 Tilled land  19 Ruderal Weed  20 Suburban / rural development  21 Continous urban  22 Inland bare ground* 18 Tilled land  19 Ruderal Weed  20 Suburban / rural development  21 Continous urban  22 Inland bare ground |
| *Grazed land* | 7 Meadow/Verge/Semi natural  5 Grass Heath  6 Mown/Grazed turf  8 Rough / Marsh Grass  9 Moorland Grass |
| *Moorland* | 10 Open shrub moor  12 Bracken  17 Upland bog  24 Lowland bog  25 Open shrub heath |
| *Open canopy forestry* | 11 Dense shrub moor  13 Dense shrub heath  14 Shrub orchard  15 Deciduous woodland  23 Felled forest |
| *Closed canopy forestry* | 16 Coniferous woodland |
| *Water bodies* | 2 Inland water |
| *Not present in this area* | 0 Unclassified  1 Sea  3 Beach and Coastal Bare  4 Saltmarsh |

*Comparison with LCM 1990*

To compare between the two land cover classifications we took a sample of 1000 random points within the study area. From these we calculated an overall agreement rate of 71.4%. To further investigate which habitat categories were most similar across the two maps we used the *crosstabs*  function in the raster (Hijmans & van Etten 2012) package in R to compare between the two land classifications cell by cell (Table 2.)90 30 90 80 30 40

**Table 2.** Confusion matrix showing percentage agreement (diagonal; **bold text**) ad disagreement (off-diagonal) between land cover categories on our classified map and LCM 1990

|  | | **Classification used in this study** | | | | | |
| --- | --- | --- | --- | --- | --- | --- | --- |
|  |  | *Human dominated areas* | *Grazed land* | *Moorland* | *Open canopy forestry* | *Closed canopy forestry* | *Water bodies* |
| **LCM 1990 classification** | *Human dominated areas* | **0** | 10 | 0 | 0 | 0 | 0 |
|  | *Grazed land* | 0 | **50** | 10 | 0 | 10 | 0 |
|  | *Moorland* | 90 80 30 40  100 | 30 | **90** | 80 | 30 | 40 |
|  | *Open canopy forestry* | 0 | 0 | 0 | **20** | 10 | 0 |
|  | *Closed canopy forestry* | 0 | 10 | 0 | 0 | **60** | 0 |
|  | *Water bodies* | 0 | 0 | 0 | 0 | 0 | **60** |

Our land cover classification was very similar, overall, to the LCM 1990 map. Some differences would, of course, be expected due to changes in vegetation and, perhaps, land use between the late eighties and 1994. The largest difference was in the areas classified as human dominated,; there was very little agreement between the two maps for this category. However, this category represents a very small proportion of the study area (around 5%) and due to the re-sampling and re-projection of these maps it is not unlikely that this category would be overlooked in places or dominated by other land cover categories. Another area where the two maps agreed less was the open canopy forestry category. This category contains several moorland scrub habitats which may indeed have been moorland when LCM 1990 was produced and have been altered by succession in the following years. In the other categories agreement was high suggesting that our classified map is a more than adequate representation of the landscape in 1994 and therefore suitable for use in our landscape simulation model.

**References**

Hijmans, R.J. & Van Etten, J. (2012) Raster: Geographic Analysis and Modeling with Raster Data. R package version 1.9-63.

R Development Core Team. (2012) R: A Language and Environment for Statistical Computing Version 2.15.0. R Foundation for Statistical Computing, Vienna, Austria. ISBN 3-900051-07-0, URL <http://www.R-project.org/>.
