# Supplementary material for: Scenario-Led Habitat Modelling of Land Use Change Impacts on Key Species
Source: PLoS One. 2015 Nov 16;10(11):e0142477. doi: 10.1371/journal.pone.0142477 (PMC4646449; doi:10.1371/journal.pone.0142477)
Supplement: S2 File — A description on the MaxEnt model used to assess habitat suitability under each scenario. (DOC) [file pone.0142477.s002.doc]

**Appendix II – Modelling black grouse habitat suitability using MaxEnt**

Here we present the full MaxEnt model used to assess habitat suitability for black grouse across the scenarios. While the landscape simulation models use only an individual run of MaxEnt the results presented here are cross-validated to give a fuller picture of habitat relationships and the results of model testing.

*Black grouse data*

Black grouse data presence data are taken from lek surveys completed by the Perthshire Black Grouse Study Group completed in 1994.

*Environmental variables*

The proportion of six habitat categories plus altitude were used to predict black grouse habitat suitability. The resolution of each of these environmental predictors was 28.5 m. The proportion of each of the six habitat types within 2 km of each pixel of the study area (Table 1) were calculated from classified Landsat images of the study area from 1994 (See Appendix I for details of validation of these classifications). Altitude data were obtained from Ordnance Survey.

*MaxEnt model and model testing*

The model was created using MaxEnt (Phillips, Anderson, & Schapire 2006) through the dismo (Hijmans *et al*. 2012) package in R (R Development Core Team 2012) using 10 crossvalidated replicates (Fig 1.). Model settings are given in Table 2. A regularisation multiplier was selected from candidate values between 1 and 19 by fitting models using each and selecting the value resulting in the lowest AIC score. MaxEnt was free to fit any of the available features types (linear, quadratic, product, threshold and hinge) were used in model fitting. Both AUC (Fielding & Bell 1997) and TSS (Allouche, Tsoar, & Kadmon 2006) were used to test the predictive accuracy of the model (Table 3.). Variable importance calculated from regularized gain over model runs is presented in Table 4. Response curves were produced for each variable independently (so that any spatial autocorrelation between predictors could not distort the variable response; Figure 2.).

**Table 1.** Percentage of each habitat type within the study area

| **Habitat type** | **Percentage of the study area** |
| --- | --- |
| Human dominated | 5.2 |
| Grazed land | 20.7 |
| Moorland | 58.2 |
| Open canopy forestry | 12.8 |
| Closed canopy forestry | 11.2 |
| Water bodies | 5.2 |

**Figure 1.** Plots of A) mean and B) standard deviation relative suitability score from 10 crossvalidated MaxEnt models predicting habitat suitability for black grouse in Perthshire, Scotland

***
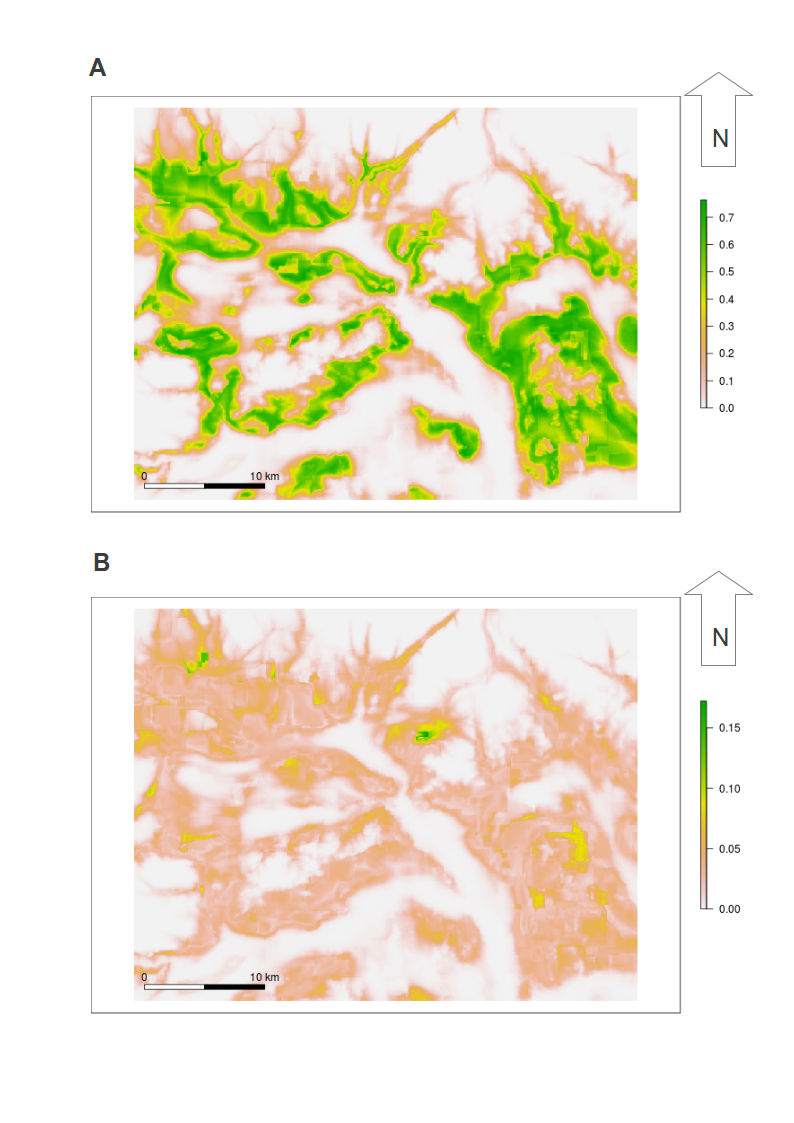
***

**Table 2.** MaxEnt settings

| Setting | Value |
| --- | --- |
| Replicated run type | Crossvalidate |
| Replicates | 10 |
| Regularization multiplier | 7 |
| Number of background points | 10000 |
| Maximum iterations | 500 |
| Convergence threshold | 0.00001 |
| Prevalence | 0.5 |

**Table 3.** Mean model test scores and standard error for MaxEnt models

| Test statistic | Mean score | S.E. |
| --- | --- | --- |
| AUC | 0.839 | 0.008 |
| TSS | 0.664 | 0.22 |

**Table 4.** Percentage contribution and permutation importance of environmental variables to the MaxEnt model

| Environmental variable | Percent contribution | Permutation importance |
| --- | --- | --- |
| Altitude | 34.5 | 56 |
| Moorland | 33 | 32.9 |
| Human dominated and rocky areas | 26.2 | 3.8 |
| Closed canopy forestry | 3.2 | 0.8 |
| Grazed land | 2.2 | 3.1 |
| Open canopy forestry | 0.6 | 2.5 |
| Water bodies | 0.2 | 0.8 |

**Figure 2.** Response curves from MaxEnt modelling for a) human dominated areas, b) proportion of grazed land, c) proportion of moorland, d) proportion of open canopy forestry, e) proportion of closed canopy forestry and f) proportion of water bodies within 2 km and g) altitude showing mean response (continuous lines) plus/minus standard error (dotted line) from ten crossvalidated models.


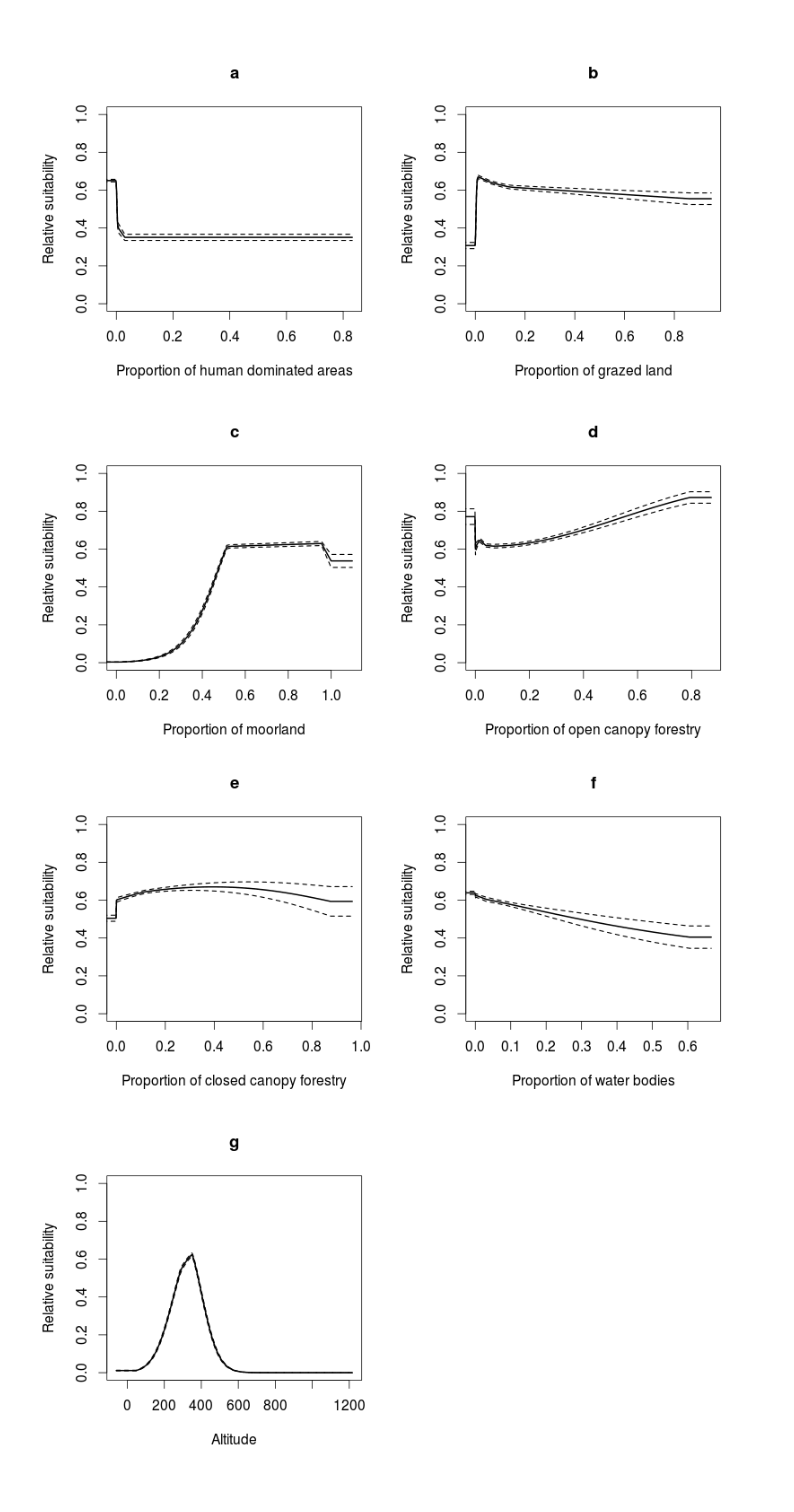


**References**

Allouche, O., Tsoar, A. & Kadmon, R. (2006) Assessing the accuracy of species distribution models: prevalence, kappa and the true skill statistic (TSS). Journal of Applied Ecology, 43, 1223–1232.

Fielding, A.H. & Bell, J.F. (1997) A review of methods for the assessment of prediction errors in conservation presence/absence models. Environmental Conservation, 24, 38–49.

Phillips, S.J., Anderson, R.P. & Schapire, R.E. (2006) Maximum entropy modeling of species geographic distributions. Ecological Modelling, 190, 231–259.

R Development Core Team. (2012) R: A Language and Environment for Statistical Computing Version 2.15.0. R Foundation for Statistical Computing, Vienna, Austria. ISBN 3-900051-07-0, URL <http://www.R-project.org/>.
