# Supplementary material for: Scenario-Led Habitat Modelling of Land Use Change Impacts on Key Species
Source: PLoS One. 2015 Nov 16;10(11):e0142477. doi: 10.1371/journal.pone.0142477 (PMC4646449; doi:10.1371/journal.pone.0142477)
Supplement: S3 File — A description of the potential scenarios indicating the scenarios chosen by experts as the most likely and the agreement between choices. (DOCX) [file pone.0142477.s003.docx]

Scenarios used in landscape simulation modelling along with how the landscape is changed under each. Scenarios were chosen by upland experts as most likely to occur in the Scottish uplands from ten candidate scenarios.

| SCENARIO | CHANGES TO THE LANDSCAPE | Rank | Percentage of experts choosing scenarios |
| --- | --- | --- | --- |
| Increased open canopy forestry | Woodland creation grants larger than those for plantation forestry are available for the planting of native forestry under the (SRDP; http://www.scotland.gov.uk/Topics/farmingrural/SRDP/RuralPriorities/Options/). This will be reflected by a conversion of 7% of grazed land and 3% of grouse moor to open-canopy/mixed woodland. Grazed land was considered more likely to be converted to woodland than grouse moor. | 1 | 86 |
| Reduced grazing | Since 1982, sheep numbers in Scotland have decreased by 34% (Scottish Government 2013). Grants encouraging a reduction in grazing are available through the Scottish Rural Development Programme (SRDP; http://www.scotland.gov.uk/Topics/farmingrural/SRDP/RuralPriorities/Options/).This reduction in upland grazing will be reflected by a 10% reduction in grazed land 7% of which will be converted into moorland and 3% into open-canopy/mixed forestry. | 2 | 57 |
| Increased grouse moor | Economic analysis of the grouse shooting industry by the Fraser of Allander Institute 2010) showed increased profitability in managed grouse shoots in 2010 and suggested this may lead to an increase in the area of moorland used for shooting. This will be reflected by a conversion of 5% of grazed land and 5% of open-canopy woodland to grouse moor. | 3 | 71 |
| Increased grazing | Both the Tenant farmers association and the Pack enquiry (Pack 2010) have suggested a return to headage payments for upland farmers. In the past, this has resulted in an increase in the number of sheep. This change will be reflected by converting 10% of moorland to grazed land. | 4 | 57 |
| Increased closed-canopy forestry | Woodland creation grants available under the (SRDP; http://www.scotland.gov.uk/Topics/farmingrural/SRDP/RuralPriorities/Options/) offer a financial incentive for the creation of plantation forestry. This will be reflected in the conversion of 5% of grouse moor and 5% of grazed land to plantation forestry. | 5 | 57 |
| Decreased grouse shooting – decrease in managed moorland, increase in grazed land | Despite a recent increase in profit from grouse shooting many managed moors do not turn a profit. This may lead to a reduction in the area of moorland managed for grouse shooting. This will be reflected by converting 6% of managed moorland to grazed land and 4% to plantation forestry. | 6 | 43 |
| Reduction in plantation forestry – Plantations converted to moorland/grazing land | Since 2005 only 65% of mature plantation forestry has been restocked after felling. To reflect this 8% of plantation forestry will be converted to grazed land and 2% to moorland. | 7 | 43 |
| Increased eco-tourism – habitat improvements to encourage wildlife | Reports on the economics of upland agriculture suggest a move by upland farmers towards more diverse sources of income and in upland areas this may involve eco-tourism such as wildlife viewing and “upland safaris”. This will be reflected by a conversion of 6% of grazed land to open canopy/mixed forestry and 4% to managed moorland. | 8 | 43 |
| Increased development – larger roads, towns and resorts | Along with UK population increases, more development in the uplands may occur as a result of increased use for leisure. This will be reflected by increasing the size of towns by 50% along with a doubling in the size of roads. Human dominated areas around loch-sides will also be increased by 10%. | 9 | 14 |
| Climate change – an increase in grassland due to drier uplands | Changing future climate may result in a drier climate for upland Scotland and moorland areas may become more grass dominated. This will be reflected by a conversion of 10% of moorland area to grazed land. | 10 | 29 |
